# A de novo missense mutation in synaptotagmin-1 associated with neurodevelopmental disorder desynchronizes neurotransmitter release

**DOI:** 10.1038/s41380-024-02444-5

**Published:** 2024-02-06

**Authors:** Maaike A. van Boven, Marta Mestroni, Petra J. G. Zwijnenburg, Matthijs Verhage, L. Niels Cornelisse

**Affiliations:** 1 Department of Functional Genomics, Center for Neurogenomics and Cognitive Research (CNCR), Vrije Universiteit (VU) Amsterdam, 1081 HV Amsterdam, The Netherlands; 2 Department of Human Genetics, Amsterdam UMC, 1105 AZ Amsterdam, The Netherlands; 3 Department of Functional Genomics and Department of Human Genetics, Center for Neurogenomics and Cognitive Research (CNCR), Amsterdam UMC-Location VUmc, 1081 HV Amsterdam, The Netherlands

**Keywords:** Neuroscience, Autism spectrum disorders, Genetics, Cell biology

## Abstract

Synaptotagmin-1 (Syt1) is a presynaptic calcium sensor with two calcium binding domains, C2A and C2B, that triggers action potential-induced synchronous neurotransmitter release, while suppressing asynchronous and spontaneous release. We identified a de novo missense mutation (P401L) in the C2B domain in a patient with developmental delay and autistic symptoms. Expressing the orthologous mouse mutant (P400L) in cultured *Syt1 null* mutant neurons revealed a reduction in dendrite outgrowth with a proportional reduction in synapses. This was not observed in single Syt1^PL^-rescued neurons that received normal synaptic input when cultured in a control network. Patch-clamp recordings showed that spontaneous miniature release events per synapse were increased more than 500% in Syt1^PL^-rescued neurons, even beyond the increased rates in *Syt1 KO* neurons. Furthermore, action potential-induced asynchronous release was increased more than 100%, while synchronous release was unaffected. A similar shift to more asynchronous release was observed during train stimulations. These cellular phenotypes were also observed when Syt1^PL^ was overexpressed in wild type neurons. Our findings show that Syt1^PL^ desynchronizes neurotransmission by increasing the readily releasable pool for asynchronous release and reducing the suppression of spontaneous and asynchronous release. Neurons respond to this by shortening their dendrites, possibly to counteract the increased synaptic input. Syt1^PL^ acts in a dominant-negative manner supporting a causative role for the mutation in the heterozygous patient. We propose that the substitution of a rigid proline to a more flexible leucine at the bottom of the C2B domain impairs clamping of release by interfering with Syt1’s primary interface with the SNARE complex. This is a novel cellular phenotype, distinct from what was previously found for other *SYT1* disease variants, and points to a role for spontaneous and asynchronous release in *SYT1*-associated neurodevelopmental disorder.

## Introduction

Information processing in the brain depends on fast neurotransmission in synapses synchronized with presynaptic action potential (AP) activity. When an AP invades the presynaptic terminal, the rapid rise in calcium activates a set of specialized proteins which catalyze the synchronous fusion of neurotransmitter-filled synaptic vesicles [1]. This core fusion machinery comprises three soluble N-ethylmaleimide sensitive factor attachment protein receptors (SNAREs), that together provide the energy for fusion. It also includes key regulatory factors involved in the assembly and activation of the SNARE complex [2]. Mutations in genes encoding components of the core fusion machinery are increasingly found to underlie neurodevelopmental symptoms in patients [3–5] and define a new class of neurodevelopmental disorders referred to as SNAREopathies [6].

In recent years, several patients with neurodevelopmental symptoms of various severity have been described with de novo heterozygous missense mutations in the gene *SYT1* [7, 8]. *SYT1* encodes the calcium sensor Synaptotagmin-1 (Syt1) and belongs to the synaptotagmin family, a group of membrane associating proteins that contain two calcium binding C2 domains [9]. Among the 17 synaptotagmins, Syt1 is the predominant paralog for fast release in the forebrain [10], involved in vesicle docking, priming, fusion and endocytosis [9, 11–15]. Syt1’s primary role is to time-lock neurotransmitter release with millisecond precision to presynaptic APs, and to prevent asynchronous and spontaneous release between APs [16–19]. Before Ca^2+^-influx, the C2B domain binds the SNARE complex through the so-called primary interface [20, 21]. This prefusion Syt1-SNARE complex is thought to hinder membrane fusion, and to keep the release machinery in a state ready for fast release. During AP-induced Ca^2+^-influx, calcium binding to the Ca^2+^-binding loops of C2B induces their insertion into the membrane (disturbing the lipid bilayer), releasing the Syt1-SNARE interaction and enabling cooperation between Syt1 and the SNAREs in membrane fusion [20, 21]. Most of the pathogenic mutations in individuals with NDDs cluster at the Ca^2+^-binding sites in the C2B domain [7, 22], although mutations in the C2A domain were also reported [8]. These C2B mutants delayed release kinetics when overexpressed in wild type neurons, and failed to support evoked release when expressed in a *Syt1 null* background [7, 22, 23]. This has led to the idea that disruption of the Ca^2+^-dependent fusion role of Syt1 is the main molecular cause of Syt1 associated developmental disorders [23].

In this paper we describe an individual presenting with developmental delay and carrying a de novo heterozygous mutation c.1202 C > T, resulting in missense variant Pro401Leu. This substitution does not locate to a calcium-binding site, but flanks the C2B bottom face involved in membrane [12, 24] and SNARE [20, 25] binding. We show that the ability of Syt1 to suppress spontaneous and asynchronous release is impaired by this mutation, while its release triggering function is not affected. This novel phenotype is distinct from cellular phenotypes found for other *SYT1* disease mutations and points towards a role for release synchronization in normal brain development.

## Results

### A novel de novo missense mutation in *SYT1* in a patient with development delay and autistic symptoms

A 5.5 years old boy visited our multidisciplinary outpatient clinic because of unexplained tall stature, autistic features, and a mild developmental delay. He was born after an uneventful pregnancy at 41 weeks gestational age and his birth weight was 5 kg. He is the second child of non-consanguineous parents with unremarkable family history. His father was also tall as a child and his current height is 196 cm (+1.7 SD). Patient’s target height is +0.84 SD. Upon physical examination the boy had a height at +2.9 SD with normal weight and head circumference. He displayed mild hypermobility of the joints. No dysmorphic features were observed, except for widely spaced teeth and persistent fetal finger pads. He had one hyperpigmentation on his arm. At the age of 2 years old, his parents noted staring episodes. An EEG was performed but no abnormalities were found. His motor development was mildly delayed, and he started walking at the age of 22 months. His speech development was limited to a few words at the age of 36 months. At his first visit he spoke full sentences. He attended regular school, but experienced learning and behavioral problems (impaired concentration, limited interaction with his peers) and currently attends special needs education. At the age of 5 years, a pervasive developmental disorder not otherwise specified was diagnosed. His total intelligent quotient (IQ) was estimated 64–81 based on intelligence tests (the Dutch Wechsler Preschool and Primary Scale of Intelligence-Third edition (WPPSI-III-NL)). In particular, sensory-processing and information-processing difficulties were identified and the child psychiatrist confirmed a neurobiological developmental disorder. At the age of 10 years he is able to walk, run and cycle. On the Vineland Adaptive Behavior Scales-3 his adaptive behavior on communication and socialization domains was measured as moderate-to-low skill level and on the daily living skills domain his adaptive skills were on an adequate-to-high level. Brain imaging as not been performed.

Compared to other cases with missense mutations in *SYT1* [7] or a deletion of *SYT1* [26] the patient described here appears to be mildly affected. The majority of these previously described cases, aged 3–21 years, had absent speech, delayed motor development, movement disorders such as dystonia or stereotypies and infant hypotonia. Their growth was normal. The phenotype of the patient described here shows overlap with these cases (delayed motor and speech development, joint hypermobility, neurobiological developmental disorder), but is remarkably milder.

The patient showed normal endocrine functions. Genetic testing revealed a normal male karyotype (46,XY), a normal repeat length of the (CGG)n repeat of the FMR1-gene, and SNP array analysis (Affymetrix CytoScan® HD) showed a normal male array pattern. Whole exome sequencing of DNA isolated from blood samples of the patient and his parents (performed with an Illumina HiSeq 4000 instrument) demonstrated a heterozygous missense variant in *SYT1* (NM_005639.2(SYT1):c.1202 C > T p.(Pro401Leu) (Chr12(GRCh37):g.79842837 C > T)). This variant was not present in his parents.

**Fig. 1:**
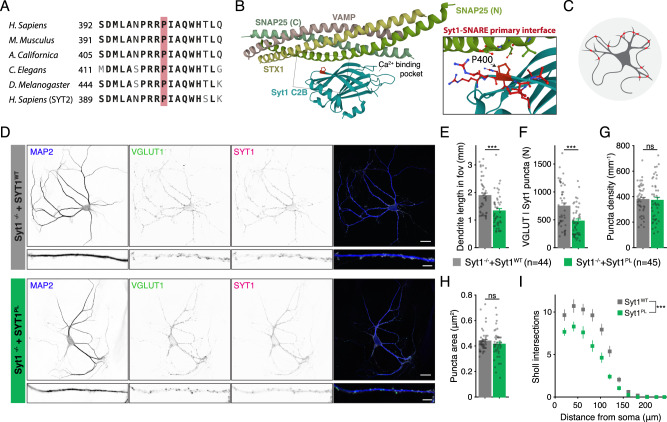
De novo missense mutation Syt1 Pro401Leu reduces dendrite length and synapse number. **A** Amino acid sequence alignment of Synaptotagmin-1 (Syt1) paralogs and main paralog Synaptotagmin-2 (Syt2). Bold letters indicate conserved residues. A red box indicates Pro401, which is conserved across the indicated sequences and corresponds to Pro400 in mouse Syt1. **B** Ribbon diagram illustrating the structure of the Syt1 C2B-SNARE complex solved by X-ray crystallography (PDB: 5KJ7 [69]). The mutated proline residue is shown in red. Inset: close-up view of the mutation site Pro400 at the Syt1-SNARE primary interface. Residues within 5 Å of Pro400 are displayed in stick representation. **C** Schematic representation of an autaptic neuron (autapse), a single neuron on a micro-island that innervates its own dendritic tree, used to investigate morphological and synaptic properties in this study. **D** Representative images of *Syt1 null* autaptic neurons rescued with Syt1^WT^ or Syt1^PL^, stained for Syt1 in addition to MAP2 and VGLUT1 as dendritic marker and synaptic marker, respectively. **E** Total dendrite length and (**F**) total number of synapses per neuron within the field of view (fov). **G** Synaptic density. **H** Average synapse size. **I** Quantification of dendritic branching by Sholl analysis. Sequence alignment was generated using the NBCI online service [70], with amino acid sequences obtained from the UniProtKB database [71]: SYT1_HUMAN (P21579), SYT1_MOUSE (P46096), SY65_APLCA (P41823), SYT1_CAEEL (P34693), SY65_DROME (P21521), SYT2_HUMAN (Q8N9I0). Scale bars: 25 μm (main panels); 5 μm (insets). Error bars represent SEM. *p < 0.05; **p < 0.01; ***p < 0.001. n is the number of neurons analyzed per condition.

The Pro401 residue is evolutionary conserved among various vertebrate and invertebrate orthologs, as well as in the human paralog Synaptotagmin-2 (Fig. 1A). No variation was found at this position in more than 76 000 genomes from healthy individuals of diverse ancestries in the gnomAD database (v3.1.2; https://gnomad.broadinstitute.org), indicating that Pro401Leu is not a common variant [27, 28]. Pro401 is located at the bottom of the C2B domain (Fig. 1B), opposite to the top Ca^2+^ binding loops, where previously characterized [7, 22, 23] mutations are located. The mutated proline is positioned next to two arginine residues (R399, R400) that directly interact with SNAP25 to form the primary Syt1-SNARE interface (inset Fig. 1B) [20, 21, 29] important for evoked synchronous release and suppression of spontaneous release [21, 29–31]. Altogether, this suggests that Pro401 could be an important residue for the function of Syt1, and that substitution with a Leucine at this location could have a pathogenic effect.

### Syt1^PL^ inhibits dendritic maturation

To investigate the effect of the patient Syt1 mutation Pro401Leu on neuronal development and synaptic transmission, we used lentiviral expression vectors containing an F2A-linked GFP (Fig. S1B) to express murine Syt1 carrying the homologous mutation Pro400Leu (Fig. 1A) (Syt1^P400L^; hereafter referred to as Syt1^PL^) or wild type Syt1 (Syt1^WT^) in cultured autaptic hippocampal neurons from *Syt1* knockout mice [32, 33]. Autaptic neurons or “autapses” are single neurons cultured on micro-islands that innervate their own dendritic tree (Fig. 1C), allowing the assessment of morphological and synaptic parameters of individual neurons in a standardized manner [34, 35]. Immunostaining revealed that expression of Syt1 and GFP were similar between Syt1^WT^ and Syt1^PL^-rescued neurons, and at the same ratio (Fig. S1A, C). Furthermore, Syt1 showed a high degree of colocalization with the presynaptic marker VGLUT1 (Fig. 1D, Fig. S1D), which was the same for Syt1^PL^ and Syt1^WT^ (Fig. S1D). Together, this indicates that that protein stability and synaptic targeting are unaffected by the PL mutation.

To assess the impact of the PL mutation on neuronal morphology, dendrites and synapses were visualized using MAP2 staining and VGLUT1/Syt1 staining, respectively (Fig. 1D) and analyzed with the semi-automated image analysis tool SynD [36]. Expression of Syt1^PL^ decreased total dendrite length per neuron by 30% (Fig. 1E), with a concomitant reduction in the number of VGLUT1^+^/Syt1^+^ synaptic puncta as compared to Syt1^WT^-rescued neurons (Fig. 1F), but had no effect on synapse density (Fig. 1G). The size of VGLUT1^+^/Syt1^+^ synaptic puncta was the same in Syt1^PL^- and Syt1^WT^-rescued neurons (Fig. 1H). Dendritic branching, as quantified by Sholl analysis, was also reduced (Fig. 1I). These results indicate that Syt1^PL^ affects dendrite outgrowth or stability, but not synaptogenesis.

### Syt1^PL^ impairs suppression of asynchronous release

**Fig. 2:**
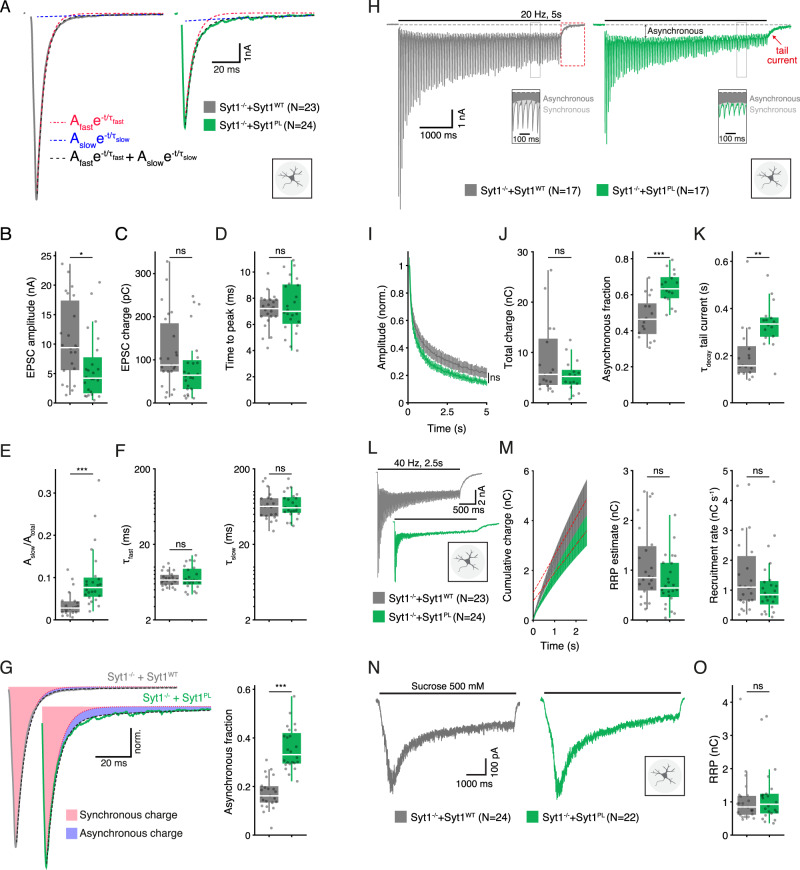
Syt1^PL^ desynchronizes neurotransmission. **A** Representative EPSC traces of *Syt1 null* autaptic neurons rescued with Syt1^WT^ or Syt1^PL^. Biexponential decay fits displayed in black dashed lines. Fast and slow components are also shown as separate mono-exponentials in red and blue, respectively. **B** EPSC amplitude. **C** EPSC charge. **D** Time interval between pulse onset and peak amplitude. **E** Relative amplitude of the slow component. **F** Fast and slow decay time constants τ_fast_ and τ_slow_. **G** Left; normalized typical EPSC traces from Syt1^WT^- and Syt1^PL^-rescued neurons with synchronous charge shown in red and asynchronous charge in blue. Right; Asynchronous fraction of total release during a single EPSC. **H** Representative traces of EPSCs during 20 Hz stimulation and tail currents after stimulation in Syt1^WT^ and Syt1^PL^-rescued neurons. The division of synchronous and asynchronous release is depicted in the inset. **I** EPSC amplitudes during 20 Hz stimulation normalized to the first EPSC in the train. **J** Total charge transferred during 20 Hz trains (left) and its asynchronous fraction (right). **K** Decay time constant of the tail current after 20 Hz stimulation. **L** Representative traces of 40 Hz stimulus trains in Syt1^WT^ and Syt1^PL^-rescued neurons. **M** Left, cumulative charge transferred during 40 Hz train, with back-extrapolated linear fits displayed in red. Right, estimated RRP and recruitment rate obtained from the Y-axis intercept and the slope of the back-extrapolated linear fits, respectively. **N** Representative traces of synaptic responses to application of 500 mM hypertonic sucrose solution and (**O**) associated RRP size estimate. *p < 0.05; **p < 0.01; ***p < 0.001. n is the number of neurons analyzed per condition.

Syt1’s main function is to trigger neurotransmitter release in response to a presynaptic AP [37]. To examine the impact of the patient mutation on AP-triggered release, we measured evoked postsynaptic currents (EPSCs) in autaptic hippocampal *Syt1 null* neurons rescued with either Syt1^PL^ or Syt1^WT^ using whole-cell voltage-clamp. The amplitude of EPSCs was considerably reduced in Syt1^PL^-rescued neurons as compared to Syt1^WT^-rescued neurons (Fig. 2A, B). Paired-pulse stimulations showed that the release probability did not differ between the groups (Fig. S2A, B). Despite a reduced amplitude, the total amount of release, as measured by EPSC charge [38] did not differ (Fig. 2C). This suggested that the time course of release was slower and more prolonged in Syt1^PL^-rescued neurons. However, rise times, measured as the time from stimulation onset to the peak of the EPSC, were the same for Syt1^PL^ and Syt1^WT^ (Fig. 2D). In order to quantify the EPSC decay time courses we fitted the decay phase with bi-exponential fits (Fig. 2A). While the decay time constants for the fast and slow components (τ_fast_ and τ_slow_) were indistinguishable (Fig. 2F), the relative amplitude of the slow component was considerably larger in EPSCs from Syt1^PL^ neurons compared to Syt1^WT^ (Fig. 2E; Fig. S2C, D). We calculated the amount of synchronous and asynchronous release during a single EPSC by: (1) adding the current integrals of the rise phase and the fast component of decay to obtain the total amount of synchronous release (Fig. S2E), and (2) integrating the EPSC’s slow decay component to obtain asynchronous release (Fig. S2F). Compared to Syt1^WT^, Syt1^PL^-rescued neurons displayed a two-fold increase in the contribution of asynchronous release to the total amount of release per EPSC (Fig. 2G). Taken together, these data show that the PL mutation increases an asynchronous component in evoked release that is almost absent in Syt1^WT^ -rescued neurons.

Syt1 also synchronizes neurotransmission by suppressing asynchronous release during prolonged stimulation [19]. To probe this function, we assessed synchronous and asynchronous release during 20 Hz stimulus trains (Fig. 2H). Rundown of synchronous EPSCs was unaffected in Syt1^PL^ neurons (Fig. 2I). In line with the results for single EPSCs, Syt1^PL^ and Sy1^WT^-rescued neurons transferred the same amount of charge during 20 Hz trains, but in Syt1^PL^, asynchronous release accounted for a larger fraction of the total release during the train (Fig. 2J). In addition, asynchronous release directly following the train (‘tail current’) was increased in Syt1^PL^ neurons, as evidenced by a slower decay to baseline (Fig. 2H, K). These data show that the PL substitution also promotes asynchronous release during, and directly after, prolonged stimulation.

We employed 40 Hz stimulus trains (Fig. 2L) to estimate the total pool of readily releasable vesicles (RRP) and the vesicle recruitment rate [39]. The total charge transferred during 40 Hz stimulation did not differ between Syt1^WT^ and Syt1^PL^-rescued neurons (Fig. S2G). Likewise, both RRP size and recruitment rate were similar in neurons expressing Syt1^PL^ and Syt1^WT^ (Fig. 2M). To corroborate our findings, we used hypertonic sucrose (500 mM) application as an independent approach to assess RRP size [35, 40] and again found no difference in RRP size (Fig. 2N, O), despite the reduced number of synapses in Syt1^PL^-expressing neurons (Fig. 1E). This suggests that Syt1^PL^-rescued neurons have more primed vesicles per synapse.

### Syt1^PL^ has a dominant-negative effect on dendrite length and suppression of asynchronous release

**Fig. 3:**
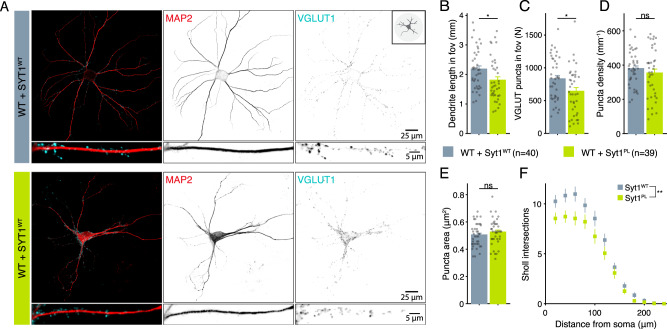
Syt1^PL^ reduces dendrite length and synapse number in the presence of endogenous Syt1^WT^. **A** Representative images of wild type (WT) neurons overexpressing Syt1^WT^ or Syt1^PL^, stained for MAP2 as dendritic marker and VGLUT1 as synaptic marker. **B** Total dendrite length and (**C**) total number of synapses within the field of view (fov). **D** Number of synapses per mm^2^ dendrite and (**E**) average synapse size. **F** Quantification of dendritic branching by Sholl analysis. Error bars represent SEM. *p < 0.05; **p < 0.01; ***p < 0.001. n is number of neurons analyzed per condition.

**Fig. 4:**
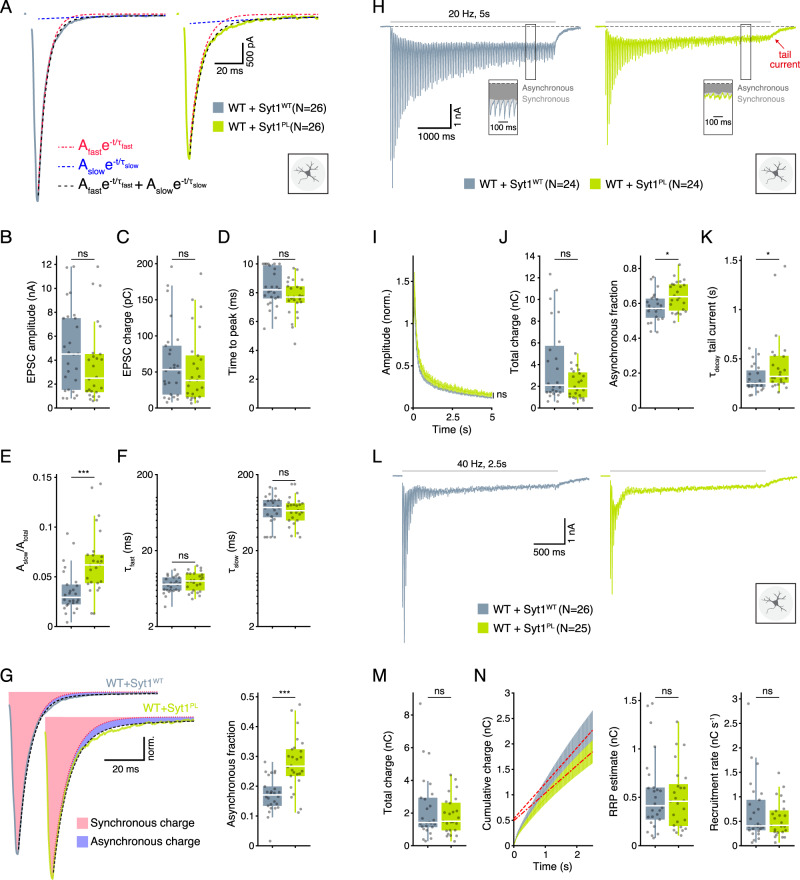
Syt1^PL^ increases asynchronous release in the presence of endogenous Syt1^WT^. **A** Representative EPSC traces of wild type (WT) neurons overexpressing Syt1^WT^ or Syt1^PL^. Biexponential decay fits displayed in black dashed lines. Fast and slow components are also shown as separate mono-exponentials in red and blue, respectively. **B** EPSC amplitude. **C** EPSC charge. **D** Time interval between pulse onset and peak amplitude. **E** Relative amplitude of the slow component. **F** Fast and slow decay time constants τ_fast_ and τ_slow_. **G** Left; normalized typical EPSC traces from Syt1^WT^- and Syt1^PL^-overexpressing neurons with synchronous charge shown in red and asynchronous charge in blue. Right; Asynchronous fraction of total release during a single EPSC. **H** Representative traces of EPSCs during 20 Hz stimulation and tail currents after stimulation in Syt1^WT^- and Syt1^PL^-overexpressing neurons. The division of synchronous and asynchronous release is depicted in the inset. **I** EPSC amplitudes during 20 Hz stimulation normalized to the first EPSC in the train. **J** Total charge transferred during 20 Hz trains (left) and its asynchronous fraction (right). **K** Decay time constant of the tail current after 20 Hz stimulation. **L** Representative traces of 40 Hz stimulus trains in Syt1^WT^- and Syt1^PL^-overexpressing neurons. **M** Total charge transferred during 40 Hz stimulation**. N** Left, cumulative charge transferred during 40 Hz train, with back-extrapolated linear fits displayed in red. Right, estimated RRP and recruitment rate obtained from the Y-axis intercept and the slope of the back-extrapolated linear fits, respectively. *p < 0.05; **p < 0.01; ***p < 0.001. n is the number of neurons analyzed per condition.

To investigate whether the Syt1^PL^ variant affects neuronal morphology and synaptic function in the presence of WT Syt1, we overexpressed Syt1^PL^ or Syt1^WT^ in wild type (WT) neurons. All morphological and electrophysiological phenotypes found on the Syt1 KO background were confirmed, although with somewhat smaller effect sizes (see Table S1). Immunostaining for MAP2 and VGLUT1 (Fig. 3A) revealed that compared to overexpression of Syt1^WT^, overexpression of Syt1^PL^ reduced dendrite length by 17% (Fig. 3B), with a proportional reduction in the number of synapses (Fig. 3C). Dendritic branching was likewise reduced (Fig. 3F), while synapse size and density were unaffected (Fig. 3D, E). EPSC amplitude, charge and time-to-peak did not differ (Fig. 4A–D). Again, time constants for the fast and slow components of the EPSC decay phase were not different (Fig. 4F), but the slow component contributed more to the total amplitude of Syt1^PL^ EPSCs (Fig. 4E, Fig. S4A, B), which was associated with a 61% increase in the asynchronous fraction of release compared to Syt1^WT^ (Fig. 4G, Fig. S4C, D). Similarly, we found an increase in the asynchronous fraction of release during 20 Hz trains, and a slower decay of the tail current after stimulation (Fig. 4H–K). Finally, the total charge released during a 40 Hz train, as well as the RRP and recruitment rate estimated from back-extrapolation, did not differ between the two groups (Fig. 4L–N). Altogether, these results indicate a dominant-negative effect of Syt1^PL^ on dendrite length and suppression of asynchronous release.

### Syt1^PL^ has a dominant-negative effect on the suppression of spontaneous miniature release events

**Fig. 5:**
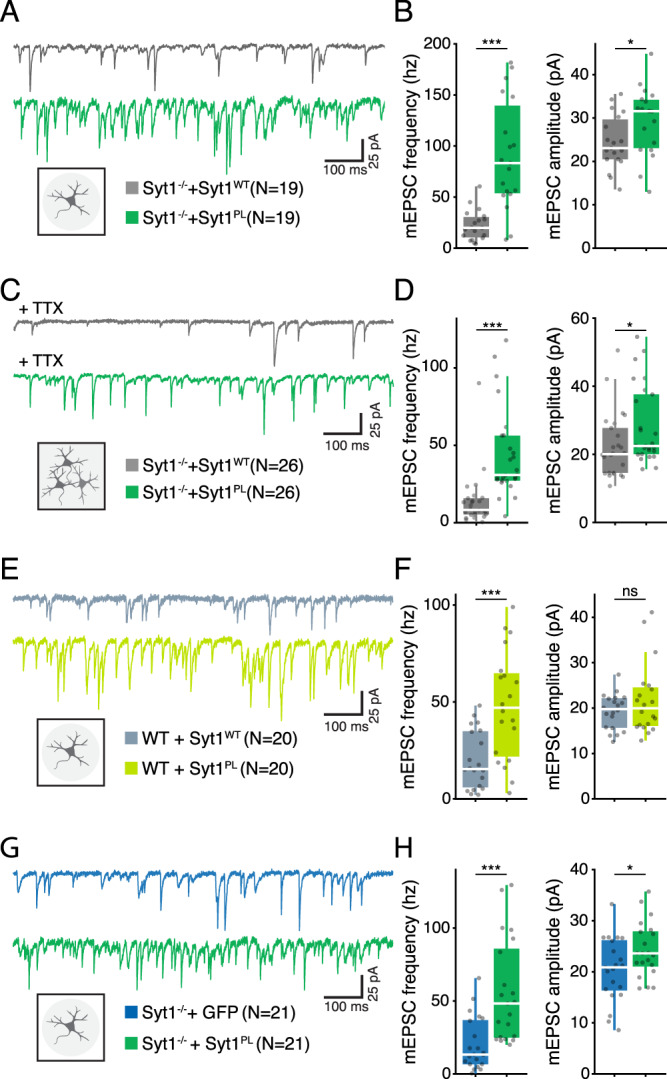
Syt1^PL^ mutation increases spontaneous transmission beyond Syt1-KO levels in autaptic- and mass- cultures. **A** Representative mEPSC recordings from autaptic hippocampal cultures of *Syt1 null* neurons rescued with Syt1^WT^ (top) or Syt1^PL^ (bottom) constructs. **B** Frequency (left) and amplitude (right) of mEPSCs in *Syt1 null* autaptic neurons rescued with Syt1^WT^ or Syt1^PL^. **C** Representative mEPSC traces recorded in TTX from hippocampal mass cultures of *Syt1 null* or Syt1 knockdown (KD) neurons rescued with Syt1^WT^ (top) or Syt1^PL^ (bottom) constructs. **D** Frequency (left) and amplitude (right) of mEPSCs recorded in TTX from Syt1^WT^ or Syt1^PL^ mass cultures. **E** Representative mEPSC recordings from autaptic hippocampal cultures of wild type (WT) neurons overexpressing Syt1^WT^ (top) or Syt1^PL^ (bottom). **F** Frequency (left) and amplitude (right) of mEPSCs in autaptic WT neurons overexpressing Syt1^WT^ or Syt1^PL^. **G** Representative mEPSC recordings from autaptic hippocampal cultures of *Syt1 null* neurons transfected with a control GFP vector (top) or Syt1^PL^ (bottom) construct. **H** Frequency (left) and amplitude (right) of mEPSCs in *Syt1 null* autaptic neurons expressing Syt1^PL^ or GFP only. *p < 0.05; **p < 0.01; ***p < 0.001. n is the number of neurons analyzed per condition.

Syt1 suppresses spontaneous vesicle fusion, preventing premature release from the RRP [16, 41, 42]. Given that the Syt1^PL^ failed to effectively suppress asynchronous release, we next examined Syt1^PL^’s ability to suppress spontaneous miniature release. Indeed, analysis of miniature EPSC (mEPSC) rates revealed a 4-fold higher rate in *Syt1 null* neurons rescued with Syt1^PL^ as compared those rescued with Syt1^WT^ (Fig. 5A, B). In addition, mEPSC amplitudes were increased in Syt1^PL^ neurons (Fig. 5B), likely due to spurious summation of independent spontaneous events at these high release rates [43, 44]. To confirm this supposition and exclude the presence of any postsynaptic effect, we repeated the experiment in younger neurons (DIV10) where mEPSC rates are still low, and found that mEPSC amplitudes were unaffected (Fig. S5), arguing against the presence of any postsynaptic effect of Syt1^PL^. When measured in mass culture in the presence of TTX (1 μM), a similar increase in mEPSC frequency was observed (Fig. 5C, D), confirming that Syt1^PL^ impairs the suppression of spontaneous miniature release and that the phenotype is not specific to autaptic cultures. Furthermore, overexpression of Syt1^PL^ on a WT background yielded a similar 3-fold increase in mEPSC release rates compared to Syt1^WT^ overexpression (Fig. 5E, F), with no differences in mEPSC amplitude (Fig. 5F). Together, these data suggest that Syt1^PL^ has a dominant-negative effect on the suppression of spontaneous miniature release.

A previous study showed that substituting the arginine residues 398 and 399 to glutamine (R398,399Q) impaired the release clamping function of Syt1 and increased the frequency of spontaneous (mEPSC) release beyond KO levels [45]. To investigate whether the P400L mutation, located immediately next to these residues, has a similar effect on spontaneous release, we compared mEPSC rates in *Syt1 null* neurons expressing either GFP only or Syt1^PL^ (Fig. 5G). *Syt1 null* neurons rescued with Syt1^PL^ exhibited a 3.5-fold increase in mEPSC frequency compared to those that only expressed GFP (Fig. 5H), with again a small effect on mEPSC amplitude (Fig. 5H). This suggests that Syt1^PL^ mutant increases spontaneous release rates beyond KO levels, probably by a mechanism similar to the Syt1 R398,399Q mutant.

### Increased spontaneous synaptic activity leads to shorter dendrites in Syt1^PL^ expressing neurons

**Fig. 6:**
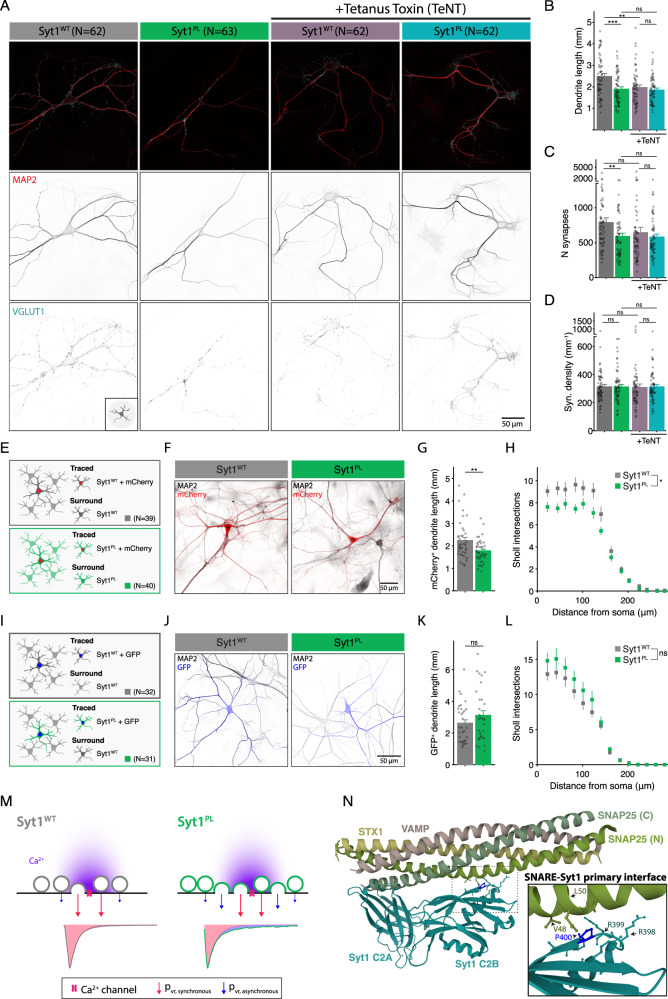
Syt1^PL^ morphological phenotype is activity-dependent. **A** Representative images of Syt1^WT^ or Syt1^PL^-rescued control (left) or tetanus toxin (TeNT)-infected neurons (right) stained for MAP2 as dendritic marker and VGLUT1 as synaptic marker. **B** Total dendrite length, (**C**) number of synapses per neuron and (**D**) synaptic density of Syt1^WT^ or Syt1^PL^-rescued neurons without and with TeNT. **E** Schematic representation of method used to trace individual neurons in Syt1^WT^- or Syt1^PL^ -rescued mass cultures by sparse infection with mCherry. **F** Representative images used for tracing of individual Syt1^WT^- or Syt1^PL^-rescued neurons that are positive for mCherry, surrounded by mCherry^−^ neurons rescued with Syt1^WT^ or Syt1^PL^, respectively, both stained with MAP2 as dendritic marker. **G** Dendrite length per Syt1^PL^- or Syt1^WT^ -rescued neuron in mass culture and (**H**) dendritic branching by Sholl analysis. **I** Schematic representation of low density seeding of Syt1^WT^- and Syt1^PL^ rescued neurons on WT networks; *Syt1 null* neurons were infected in suspension with Syt1^WT^-F2A-GFP or Syt1^PL^-F2A-GFP, and plated at low density together with WT neurons from littermates. **J** Representative images used for tracing, with isolated Syt1^WT^ or Syt1^PL^-rescued neurons that are positive for GFP, surrounded by GFP^−^ WT neurons, both stained with MAP2 as dendritic marker. **K** Total dendrite length per Syt1^PL^- or Syt1^WT^ -rescued neuron and (**L**) dendritic branching by Sholl analysis. **M** Working model for neurotransmitter release from Syt1^WT^ and Syt1^PL^ expressing synapses. The arrival of an action potential (AP) in the synaptic terminal induces a strong influx of Ca^2+^ (in purple) which reaches high concentrations in the immediate vicinity of the Ca^2+^ channel but rapidly decays to lower concentrations at further distances. The vesicular release probability of vesicles in close vicinity to the channel (p_vr,synchronous_) is the same in Syt1^WT^ and Syt1^PL^ expressing synapses, since the mutation does not change Syt1’s release-triggering function. However, impaired clamping of release in Syt1^PL^-expressing synapses leads increases spontaneous release and renders vesicles more sensitive to lower Ca^2+^ concentrations further away from the Ca^2+^ channel, and for a longer duration after the AP, increasing the vesicular release probability for asynchronous release (p_vr,asynchronous_). Together with an increased RRP per synapse this results in increased asynchronous release in response to AP stimulation. **N** Crystal structure (PDB: 5CCH [21]) of Syt1-SNARE interface (murine) showing mutated Pro400 in blue. Inset: close-up view of Pro400 and surrounding (<5 Å) residues, that make up region II of the primary interface [21]. Pro400 is located next to two arginines (Arg398, Arg399), which interact with the SNARE complex and are important for clamping of release. Substitution of a rigid proline to a more flexible leucine at this position could affect the correct positioning of the arginines, thereby impairing their clamping ability. Pro400 is directly opposite of the residues Val48 and Leu50 in SNAP25, both implicated in NDDs. *p < 0.05; **p < 0.01; ***p < 0.001. n is the number of neurons analyzed per condition.

The marked increase in spontaneous release rates caused by Syt1^PL^ is hard to reconcile with the reduction in total synapse number caused by this mutation. This prompted us to wonder whether the morphological effects are caused by a compensatory mechanism to normalize spontaneous release rates, or vice versa. To address this question, we analyzed the morphology of *Syt1 null*/Syt1 knockdown (KD) neurons rescued with either Syt1^PL^ or Syt1^WT^ in the presence and absence of tetanus neurotoxin (TeNT), which silences all synaptic vesicle release by cleaving the essential SNARE VAMP2 [46]. The difference in dendrite length and synapse number observed between Syt1^PL^ or Syt1^WT^ expressing neurons was abolished in the presence of TeNT, while synaptic density was unchanged for all conditions (Fig. 6A–D). This suggests that the morphological phenotype is driven by synaptic activity, rather than a cell-autonomous effect. Interestingly, post-hoc analysis indicated that TeNT also reduced dendrite length (Fig. 6B), but not the number of synapses (Fig. 6C) in Syt1^WT^ -rescued neurons, possibly due to the complete silencing of synapses, including AP-triggered neurotransmission.

To further investigate the role of spontaneous release on neuronal morphology, we tested whether receiving synaptic input from Syt1^WT^ neurons could rescue the morphology of Syt1^PL^-expressing neurons. First, we assessed whether Syt1^PL^ reduced dendrite length in mass cultures by tracing sparsely labeled single neurons in the network (Fig. 6E, F). Indeed, we found a reduced dendrite length in Syt1^PL^ mass cultures (Fig. 6G, H), demonstrating that both the electrophysiological (Fig. 5C, D) and morphological phenotype are not restricted to autaptic cultures, but also present in mass cultures. Next, we co-cultured *Syt1 null* neurons rescued with either Syt1^PL^ or Syt1^WT^ at a ratio of ~1:8 with WT neurons from littermates, assuming that the rescued neurons would predominantly receive spontaneous input at normal frequencies from the surrounding WT neurons (Fig. 6I). In this preparation, the dendrite lengths of Syt1^PL^-rescued neurons were similar to those of Syt1^WT^-rescued neurons (Fig. 6J, K), and we found no difference in dendritic branching (Fig. 6L). These finding suggest that the observed reduction in dendrite length and dendritic branching exhibited by Syt1^PL^ autaptic neurons is not cell-autonomous, but might rather depend on altered synaptic input.

## Discussion

### Syt1^PL^ selectively impairs clamping of spontaneous and asynchronous release

In this study we identified a missense de novo mutation at a highly conserved location in Syt1 (P401L) in a patient with developmental delay and ASD symptoms. The PL mutant was equally well expressed and targeted to synapses as was WT Syt1. This suggests that the mutation causes disease by specific functional deficits, rather than protein instability/degradation or targeting deficits. *Syt1 null* neurons rescued with Syt1^PL^ showed a 30% reduction in dendrite length with a concomitant reduction in synapses. The amount of asynchronous release per synapse during a single AP or train stimulation was increased, and spontaneous miniature release events were drastically increased in these neurons. The overall physiological and morphological phenotype observed with Syt1^PL^ on *Syt1 null* background was also observed when this variant was expressed in WT neurons. Therefore, we conclude that this mutation causes disease due to a dominant-negative effect on Syt1 function.

While the increase in asynchronous and spontaneous release points to a presynaptic effect of Syt1^PL^, synaptotagmins and SNAREs also have a postsynaptic role in membrane insertion of AMPA receptors (AMPARs) [47, 48]. One possibility is that increased spontaneous AMPAR exocytosis is responsible for the increased mEPSC amplitude in Syt1^PL^-rescued neurons. An alternative explanation is the summation of simultaneous occurring mEPSC due to the high spontaneous release rates in this group. Indeed, in younger neurons with much lower mEPSC frequencies no effect on mEPSC amplitude was found, making the latter explanation more likely.

The effects on dendrite length and synapse number could arise either from a cell-autonomous postsynaptic effect of Syt1, or be induced by changes in synaptic input. The morphological differences between Syt1^PL^ and Syt1^WT^ neurons disappeared when synaptic activity was silenced by TeNT. In addition, dendrites of Syt1^PL^-rescued neurons developed normally when embedded in a mass culture of wild type cells, presumably receiving normal synaptic input from neighboring neurons, but not when receiving increased spontaneous input in Syt1^PL^ mass culture. These findings suggest that the morphological changes are secondary to the electrophysiological phenotype. We speculate that this could be a homeostatic adaptation to the exceptionally high spontaneous miniature release rates and/or to increased AP-induced total release per synapse in these neurons [49], since no morphological defects were reported for Syt1 knockout (KO) neurons [34].

In contrast to previously described *SYT1* disease variants (see below), the PL mutation did not affect the Ca^2+^-dependent fusion triggering function of Syt1. The fast component of AP-evoked EPSC’s showed normal kinetics, and normal short-term synaptic plasticity during train- or paired-pulse stimulation implied a normal vesicular release probability. Interestingly, despite the 30% reduction in synapses in Syt1^PL^-rescued neurons, we found a similar total RRP for the WT and PL group, suggesting an increased primed pool per synapse in Syt1^PL^-rescued neurons. Since both EPSC amplitudes and the total number of synapses were smaller in these neurons, these extra primed vesicles appear not to contribute to synchronous release, presumably docking more distantly from Ca^2+^ channels. Indeed, asynchronous release in response to a single AP or train stimulation was much more pronounced in Syt1^PL^-rescued synapses. In addition, we found that asynchronous release following train stimulation decayed slower back to baseline, further suggesting that clamping of asynchronous release is impaired by this mutation. In line with this notion, the mEPSC frequency was increased 4-fold in Syt1^PL^ expressing neurons. Taking into account the reduced number of synapses, this translates to a 6-fold increase in spontaneous release from individual synapses. Altogether, our observations are best explained by a model in which Syt1^PL^ increases the RRP for asynchronous release and has a selective impairment in the clamping of spontaneous and asynchronous release, while its release triggering function remains intact. In such a scenario, vesicles in Syt1^PL^ and Syt1^WT^-rescued synapses will respond similarly to peak Ca^2+^ during single APs, and release with the same probability when located close to Ca^2+^ channels (Fig. 6M). However, reduced clamping in Syt1^PL^-rescued synapses renders vesicles more susceptible to low Ca^2+^ concentrations that occur locally immediately after the AP, or globally during train stimulation, hence increasing the probability for these vesicles to be released asynchronously.

### Syt1^PL^ has a unique cellular phenotype compared to other Syt1 disease mutations

Previously, physiological phenotypes of other de novo mutations in *SYT1*-associated with neurodevelopmental disorder [7, 22] have been studied. These mutations, mostly located in the Ca^2+^-binding and membrane penetrating loops of the C2B domain (D304G, D366E, and I368T) caused an almost complete loss of AP-induced synchronous release, while the ability to clamp spontaneous release varied between the genotypes [23]. The phenotype that we found for the current Syt1^PL^ patient mutation, characterized by specific increases in spontaneous and asynchronous release without affecting synchronous release, is very dissimilar to those described for the C2B mutations. Spontaneous release has been proposed to play a role in guiding synapse maturation during development, synaptic scaling, homeostatic plasticity and excitability of neuronal networks [50, 51]. Furthermore, desynchronization of release could negatively impact the precise timing of neurotransmission, important for spike-timing dependent plasticity and information processing [52, 53]. As such, the mutation in the patient could potentially affect these aspects of brain function. We further show that dendritic growth was inhibited in the Syt1^PL^-rescued neurons. Abnormalities in dendritic growth have been proposed to be some of the major defects in ASD [54, 55], and might be causal to suggested differences in long-range versus short-range connections in ASD [56, 57]. While several cell models for neurodevelopmental disorders display either shortened [6, 58–62] or elongated [58, 63, 64] dendrites, dendritic abnormalities are absent in Syt1 KO neurons [34], and have not been associated with Syt1 disease mutations reported so far [7, 22, 23]. These findings demonstrate that mutations in the same synaptic gene can cause different cellular phenotypes, but largely overlapping clinical symptoms.

### Perturbation of the SNARE-Syt1 primary interface impairs clamping function of Syt1

Here we report a point mutation in Syt1 that selectively impairs its clamping function and increases priming. Decoupling of the clamping and activation roles was shown before for the experimental Syt1^9PRO^ mutant, but with a more drastic modification of Syt1, involving the insertion of nine prolines in the linker between the C2A and C2B domain [14, 19]. The mutated proline P401 described in the current study is at a location that forms Syt1’s primary interface with the SNARE complex [21], and is directly adjacent to two arginine residues (R399, R400 in human Syt1; R398, R399 in mouse Syt1) (Fig. 6N). These arginines protrude from the bottom of the C2B domain to interact with SNAREs at rest, most likely to prevent full zippering, thereby preventing premature neurotransmitter release [20]. Previous studies showed that mutating the positively charged arginines to neutral glutamines (Syt1^RQ^) impaired the release clamping function, and increased spontaneous release rates beyond Syt1 KO levels [24, 45], similar to the effects of the PL mutation in the current study. These exceptionally high spontaneous release rates have been attributed to the C2A domain, still present in the Syt1^RQ^- (and Syt1^PL^-) expressing neurons, but not Syt1 KO neurons, which promotes spontaneous release in the absence of the release clamping action of the C2B domain [45]. However, contrary to what we found for the PL mutation, vesicle docking was strongly reduced, and synchronous release was completely abrogated in the RQ mutant [12, 24, 45]. We speculate that the specific loss of clamping in the PL mutant originates from rendering the primary interface less rigid through the substitution of a rigid proline with a more flexible leucine [65]. This could hamper the correct positioning of the arginines in the Syt1-SNARE interaction necessary for release clamping, while they are still available for membrane binding during docking [12] and evoked synchronous release [66].

Interestingly, the Syt1^PL^ synaptic phenotype shows strong similarities to some of the recently described NDD-associated mutations located in the domain of SNAP25 that forms the primary interface with Syt1 [21, 50]. In particular, the SNAP25 variants L50S and V48F, both of which are located exactly opposite to Syt1 P401 in the crystal structure [21, 29] (Fig. 6N) strongly augmented spontaneous release with mild or no impairments in evoked release [50]. Hence, mutations in different genes that are part of the same complex can produce similar cellular phenotypes, whereas different mutations in the same gene can produce different cellular phenotypes (see discussion above). This shows the importance of in vitro phenotyping to uncover disease mechanisms in patients with different mutations in presynaptic genes.

## Methods and materials

### Ethical statement

Consent to describe the patient was obtained from the parents and apart from sequencing, no data was collected directly from the patient. Animals were housed and bred according to institutional and Dutch governmental guidelines, and all procedures are approved by the ethical committee of the Vrije Universiteit, Amsterdam, The Netherlands.

### Laboratory animals and primary neuron cultures

Syt1^−/−^ mice were generated by interbreeding heterozygous mice (C57BL/6 background). Hippocampal autaptic- and mass cultures were prepared as previously described [67]. In a few experiments Syt1 knockdown (KD) neurons were used, which is indicated in the text and figure legends. A detailed description of the culture methods and infection is given in the supplementary information.

### Morphological analysis

Morphological parameters were obtained from confocal images of autaptic or mass cultures stained for dendritic (MAP2) and synaptic markers (Syt1 and VGLUT) and analyzed using the automated image analysis routine SynD [36]. A detailed description of the imaging and analysis methods is provided in the supplementary information.

### Electrophysiology

Whole-cell voltage clamp recordings were performed in autaptic and mass cultures to measure evoked and spontaneous mEPSC release. Hypertonic sucrose application was used to estimate the readily releasable pool. All recordings were analyzed with in-house developed Matlab scripts. A detailed description of the patch-clamp experiments and analysis is provided in the supplementary information.

### Statistical analysis

Statistical significance was determined using student’s- or Welch’s *t* tests, and Mann-Whitney *U* tests when normality could not be assumed. An overview of all statistical analyses and summary statistics (mean ± SD, or median ± MAD (median absolute deviation [68]) in case of deviations from normality) is provided in Table S1. All statistical tests were two-tailed, and *p* values below 0.05 were considered significant. A detailed description of the statistical methods is provided in the supplementary information.

## Supplementary information

Table S1

Supplementary material
