# Supplementary material for: A de novo missense mutation in synaptotagmin-1 associated with neurodevelopmental disorder desynchronizes neurotransmitter release
Source: Mol Psychiatry. Author manuscript; Available in PMC 2024 Sep 5. (PMC11371641; doi:10.1038/s41380-024-02444-5)
Supplement: Supplementary material [file EMS194815-supplement-Supplementary_material.doc]

Supplementary information

# A *de novo* missense mutation in *synaptotagmin-1* associated with neurodevelopmental disorder desynchronizes neurotransmitter release.

Maaike A. van Boven^1^, Marta Mestroni^1^, Petra J.G. Zwijnenburg^2^, Matthijs Verhage^1,3^, L. Niels Cornelisse^3^

^1^Department of Functional Genomics, Center for Neurogenomics and Cognitive Research (CNCR), Vrije Universiteit (VU) Amsterdam, 1081 HV Amsterdam, The Netherlands.

^2^Department of Human Genetics, Amsterdam UMC, 1105 AZ, Amsterdam, The Netherlands.

^3^Department of Functional Genomics and Department of Human Genetics, Center for Neurogenomics and Cognitive Research (CNCR), Amsterdam UMC-Location VUmc, 1081 HV Amsterdam, The Netherlands.

Contents:

Supplementary figures S1-S6

Supplementary methods and materials

**
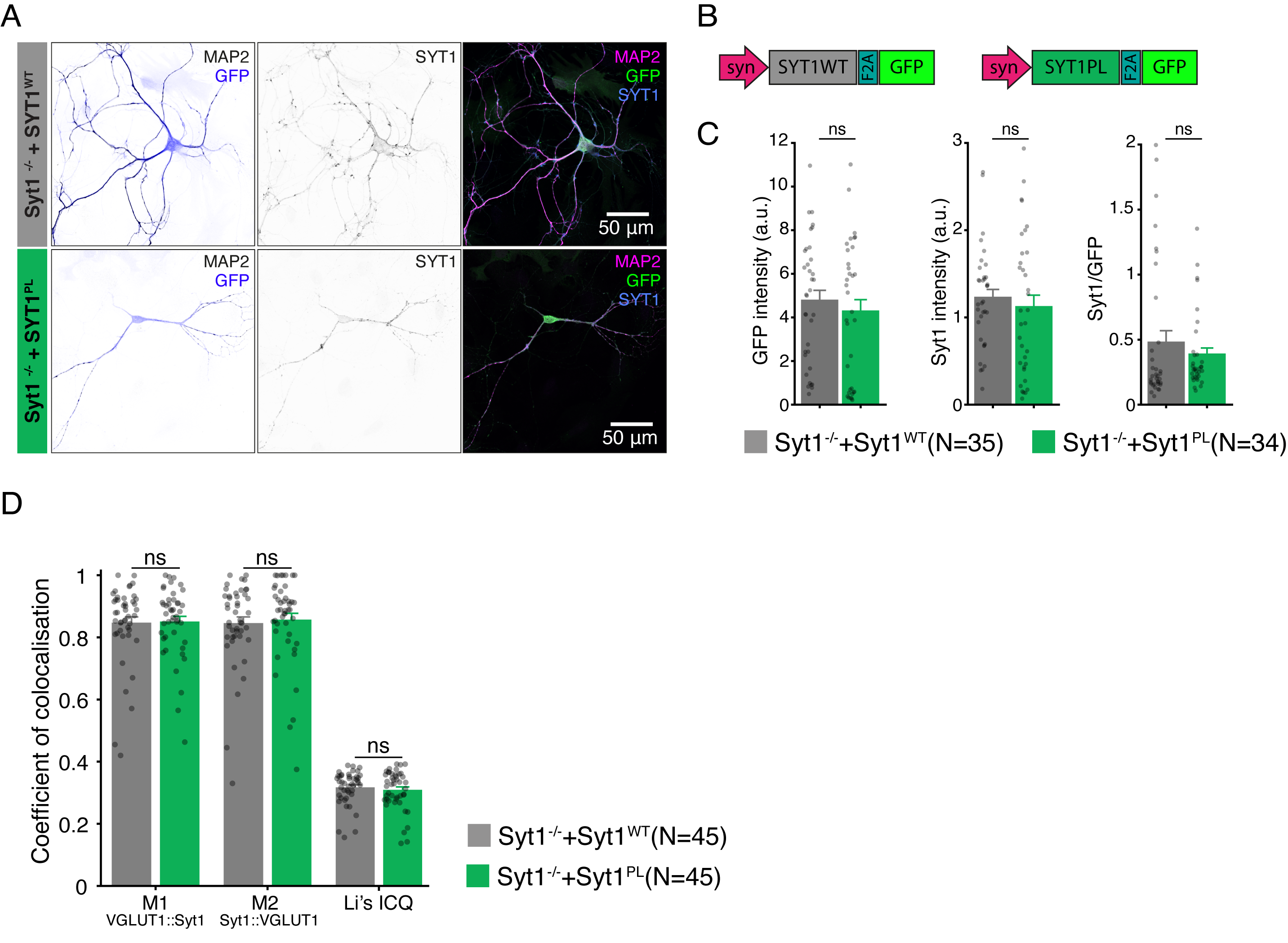
**

**Figure S1 Syt1^PL^ is stably expressed and properly targeted to synapses
(A)** Representative images of *Syt1 null* neurons rescued with Syt1^WT^ or Syt1^PL^, stained for MAP2, GFP and Syt1. **(B)** Construct diagrams of lentiviral expression vectors, in which Syt1^WT^ (left) or Syt1^PL^ (right) is linked to GFP via a self-cleaving F2A sequence in order to ensure expression of Syt1 and GFP at stable ratios. **(C)** Average GFP intensity, Syt1 intensity and the ratio of Syt1/GFP intensities in Syt1 null neurons rescued with the Syt1^WT^ or Syt1^PL^ constructs shown in panel B. **(D)** Coefficients of colocalization Manders’ M1 (Syt1 in VGLUT1), M2 (VGLUT1 in Syt1), and Li’s intensity correlation quotient (ICQ). Error bars represent SEM. N is the number of neurons analyzed per condition.

**
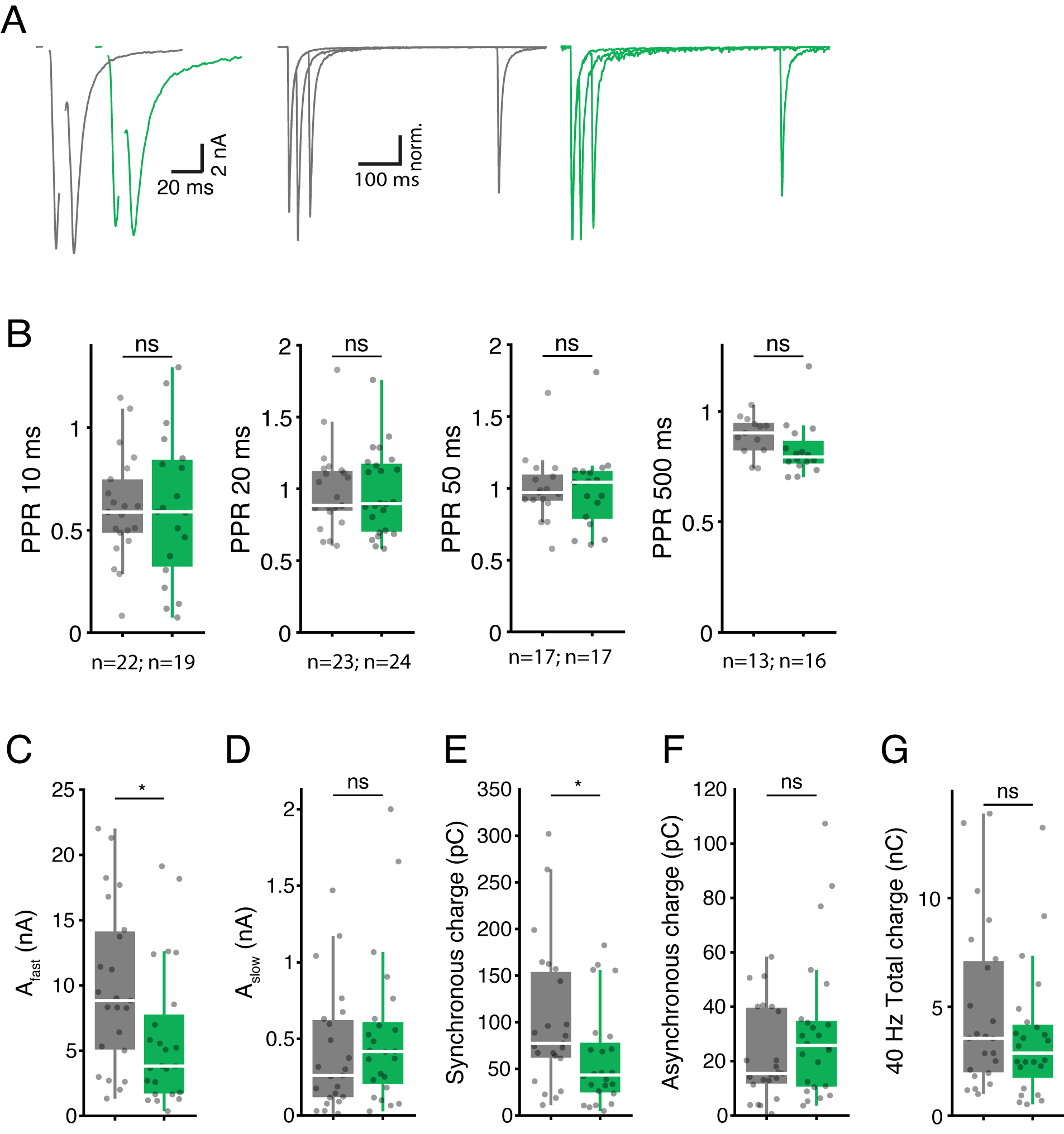
**

**Figure S2. Paired-pulse ratios of *Syt1 null* neurons rescued with Syt1^WT^ or Syt1^PL^ and absolute values for biexponential fits of EPSCs in Fig 2
(A)** Representative traces showing paired pulse stimulation at inter-pulse intervals 10 ms (left) and 20 ms, 50 ms and 500 ms (right). **(B)** Boxplots of paired-pulse ratios at 10 ms, 20 ms, 50 ms and 500 ms. **(C-D)** Absolute values of amplitudes of **(C)** the fast component A_fast_ and **(D)** slow component A_slow_ of the biexponential fit of the EPSC in Fig 2A. **(E-F)** (Related to figure 2G) Absolute values of synchronous **(E)** and asynchronous **(F)** charge transferred during single EPSCs. **(G)** (Related to Fig 2L, M) Total charge transferred during 40 Hz stimulation. *, p < 0.05.

**
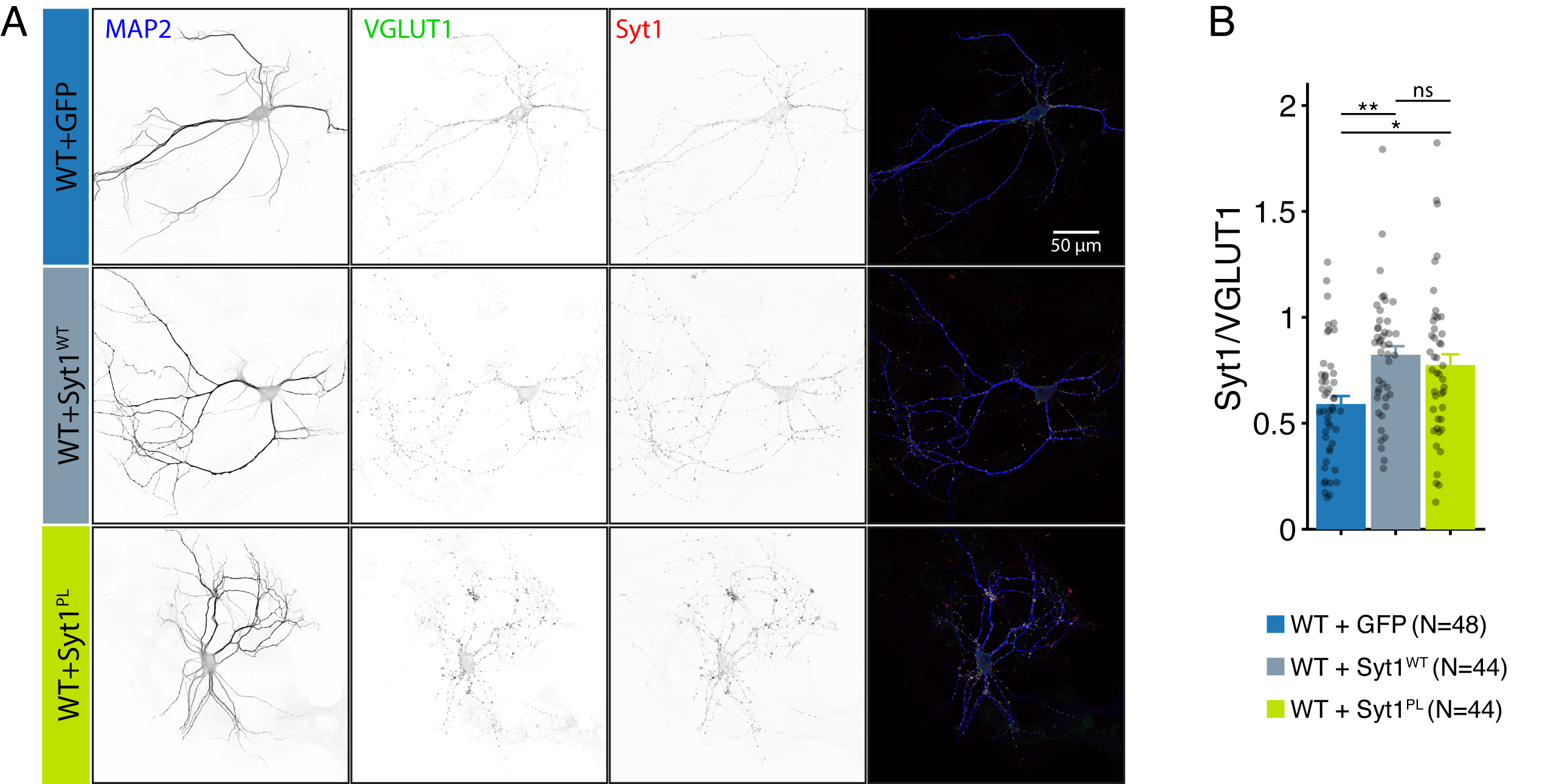
**

**Figure S3. Quantification of the degree of overexpression of Syt1^WT^ and Syt1^PL^**

**(A)** Representative images of wild type (WT) neurons expressing GFP as control, or overexpressing Syt1^WT^ or Syt1^PL^, stained against MAP2, VGLUT and Syt1. **(B)** Quantification of Syt1 intensity normalized against VGLUT1 measured in WT neurons expressing GFP (control), or WT neurons overexpressing Syt1^WT^ or Syt1^PL^. *, p < 0.05; **, p < 0.01.

**
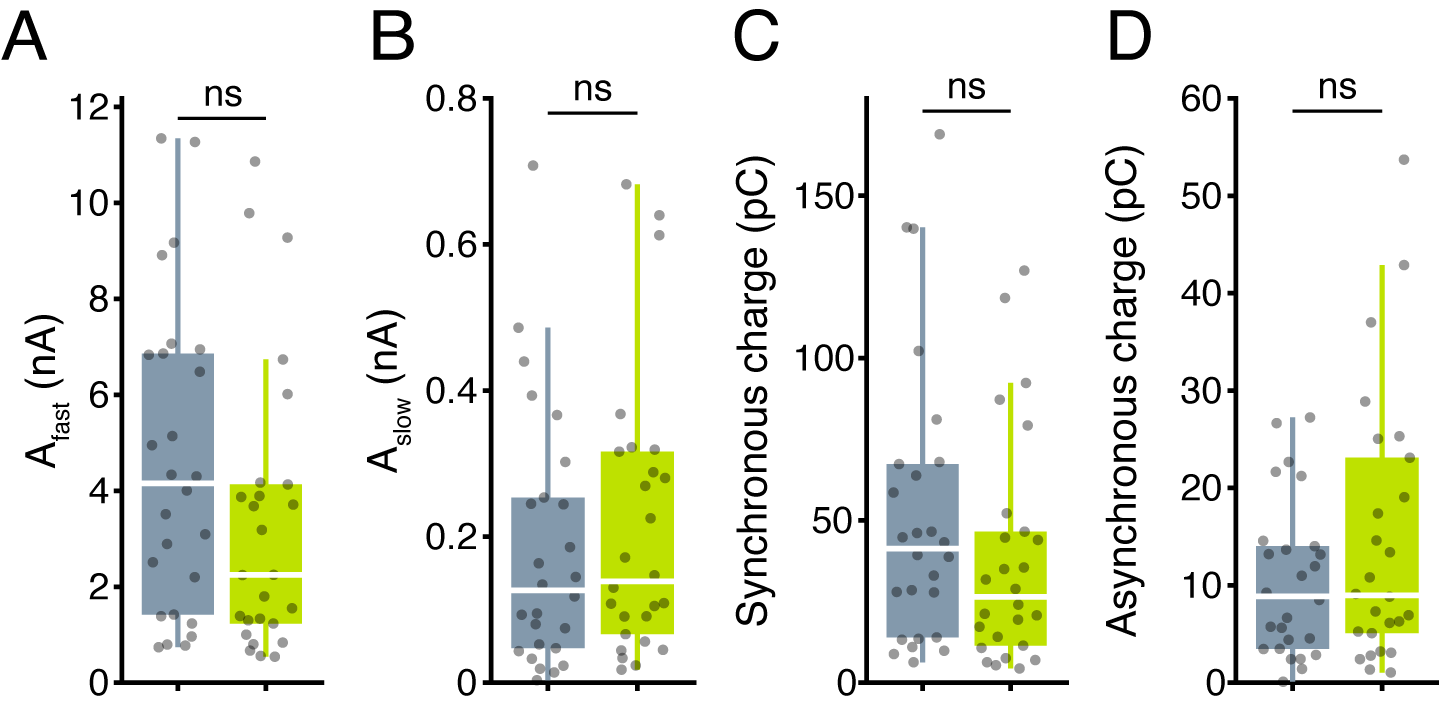
**

**Figure S4. Absolute values for biexponential fits of EPSCs in Fig 4
(A)** Absolute values of amplitudes of the fast component A_fast_ and **(B)** slow component A_slow_ of the biexponential fit of the EPSC in Fig 4A. **(C)** (Related to figure 4G) Absolute values of synchronous and **(D)** asynchronous charge transferred during single EPSCs.


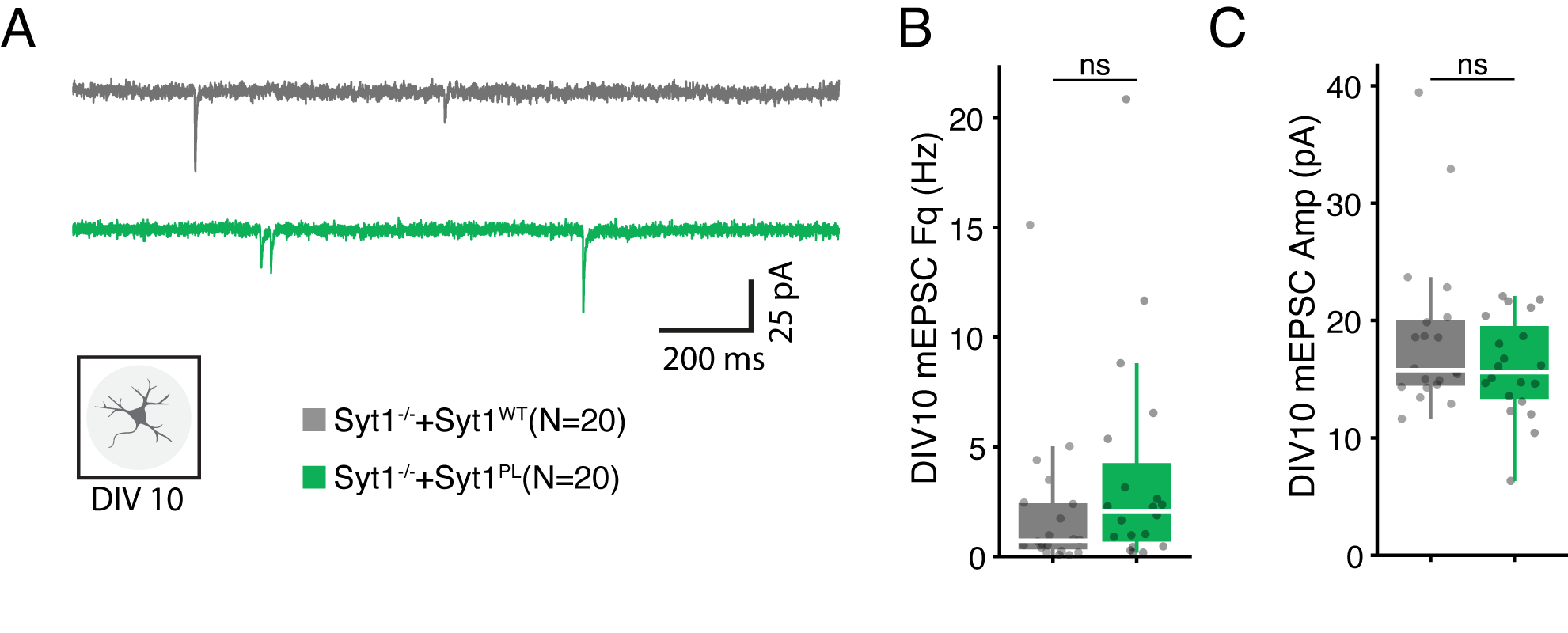


**Figure S5. Quantification of mEPSC frequency and amplitude in young neurons (DIV10)**

**(A)** Representative mEPSC recordings at DIV10 from autaptic hippocampal cultures of Syt1^WT^ (top)- or Syt1^PL^ (bottom)-rescued neurons**. (B-C)** mEPSC frequency **(B)** and mEPSC amplitude **(C)** recorded at DIV 10 in Syt1 KO/Syt1 KD neurons rescued with Syt1^WT^ or Syt1^PL^.

**
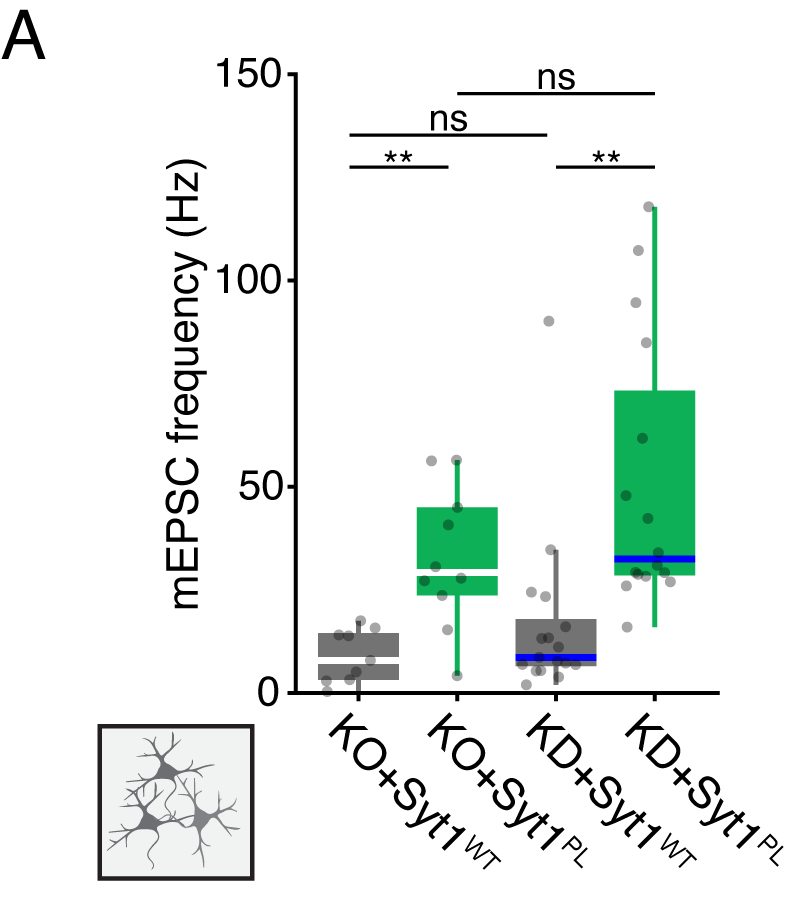
**

**Figure S6. The mEPSC frequency phenotype of Syt1^PL^ does not differ between Syt1 knockout (KO) and Syt1 knockdown (KD) rescued neurons. (A)** Boxplot displaying mEPSC frequencies in mass culture of Syt1 KO neurons rescued with Syt1^WT^ or Syt1^PL^ and Syt1 KD neurons rescued with Syt1^WT^ or Syt1^PL^. **, p < 0.01.

Supplementary Methods & Materials

***Laboratory animals and primary neuron cultures***

Syt1^−/−^ mice were generated by interbreeding heterozygous mice (C57BL/6 background). Hippocampi from embryonic day 18 (E18) pups of both sexes were dissected in Hank’s Balanced Salt Solution (HBBS, Sigma) buffered with 10 mM HEPES (Gibco), digested in 0.025% Trypsin (Gibco) for 20 min at 37 °C, triturated with a fire-polished Pasteur pipette, and resuspended in Neurobasal medium supplemented with 2% B27, 1.8% HEPES, 0.25% GlutaMAX, and 0.1% Penicillin-Streptomycin (NB+; all Gibco). Dissociated neurons were plated at a density of 1.2-1.5k per well on astrocyte micro-island coverslips. Micro-island cultures were prepared by plating 4-5k rat glial cells on agarose-coated 18-mm glass coverslips that were stamped with 0.1 mg/ml poly-D-lysine (sigma) and 0.7 mg/ml rat tail collagen (Corning) in 10 mM acetic acid (Sigma) to create 250 μm-diameter dots, as described previously^1^. For mass cultures (Fig 5C, D, Fig 6E-L), rat glial cells were plated at 25k/well on 18-mm glass coverslips coated with 0.5 milli-percent poly-L-ornithine and 2.5 μg/ml laminin (both from Sigma). All neuronal cultures were maintained in NB+ at 37°C in a humidified incubator with 5% CO_2_.

For rescue and overexpression experiments, autaptic neurons were transfected at DIV 4 with a synapsin-promoter-driven lentiviral vector expressing either murine Syt1^PL^ (Syt1^P400L^) or murine wild type Syt1 (Syt1^WT^), linked to GFP via an auto-cleaving F2A peptide sequence (Fig S1B), or with synapsin-driven GFP for the experiment described in Fig 5G, H. The degree of overexpression did not differ between Syt1^WT^ and Syt1^PL^ and final Syt1 levels were ~35% higher than endogenous Syt1 levels in wild type neurons (Fig S3). Rescue experiments were done on a Syt1 knockout background, except for the experiments in panel 2N-O, 5C,D, 6A-H and S5, which include rescue of Syt1 KO and Syt1 knockdown (KD) neurons. There was no difference in the phenotype of Syt1^PL^ between Syt1KO and Syt1KD-rescued cells (Fig S6). For Syt1 KD-based rescue experiments, neurons were infected at DIV 2 with anti-Syt1 shRNA described previously^2^, kindly provided by Thomas Südhof (Howard Hughes Medical Institute, Stanford, CA, USA).

For the experiments described in Fig6E-L, Syt1^−/−^ neurons were incubated with Syt1^PL^-F2A-GFP or Syt1^WT^-F2A-GFP (Fig 6I-L), or with mCherry (Fig 6E-H) -containing lentiviral solutions in NB+ in conical tubes at 37 °C. After 2.5-3h, the cells were washed three times with DMEM, resuspended in NB+ and plated at 2-3.5k together with 25k uninfected neurons. For tracing of single cells in mass culture in Fig 6E-H, all neurons were subsequently infected with Syt1^WT^ or Syt1^PL^. To express tetanus toxin (TeNT) (Fig6A-D), neurons were infected at DIV 0 with a previously described^3^ lentiviral vector encoding TeNT-IRES-mCherry under the synapsin promotor.

**Morphological analysis**

Neurons were fixed at DIV 17 using 3.7% formaldehyde (Electron Microscopy Sciences) in PBS for 20 minutes at room temperature. Cells were permeabilized with 0.5% Triton X-100 for 5 min and blocked with 2% normal goat serum (NGS, Life Technologies) in 0.1% Triton X-100 for 45-60 minutes. Cells were incubated overnight at 4°C with primary antibodies (chicken anti-MAP2 (1:350, Abcam ab5392); mouse anti-Syt1 (1:175, SySy #105 001) and rabbit anti-VGLUT1 (1:500, SySy #135 302) or rabbit anti-GFP (1:500, Abcam ab290) in 2% NGS and 0.1% Triton X-100. After washing with PBS, cells were incubated for 1 h at room temperature with secondary antibodies conjugated to Alexa dyes (1:1000, Invitrogen Molecular Probes), washed again and mounted with DABCO-Mowiol (Invitrogen). Images were acquired at 2048x2048 pixels on a Nikon Eclipse Ti confocal laser scanning microscope (40× objective; NA 1.3) with NIS-Elements 4.30 software. Zoom was 1.12 for the autapse dataset and 1.0 for the mass culture dataset. Confocal settings were kept constant for all scans within an experimental week. Fiji^4^ was used to create maximum-intensity projections of 3 z-planes, and to perform colocalization analysis using the coloc_2 plugin. Analysis of neuronal morphology was performed using the automated image analysis routine SynD^5^. Experimental conditions were blinded during analysis. Exclusion criteria were: blurry image, damaged dendritic tree and absence of VGLUT puncta.

**Electrophysiology**

*Patch-clamp recordings.* Whole-cell voltage-clamp recordings were obtained from DIV13-17 neurons using borosilicate glass pipettes (tip resistance: 2.5-4.5 MΩ) filled with (in mM): 125 K-gluconate, 10 NaCl, 4.6 MgCl_2_, 4 ATP- K_2_, 15 Phosphocreatine, 1 EGTA and 10 units/mL creatine phosphokinase, pH adjusted to 7.3 and osmolarity to 290 mOsm. The extracellular solution (ACSF) contained (in mM) 140 NaCl, 2.4 KCl, 2 CaCl_2_, 4 MgCl_2_, 10 HEPES and 10 glucose, pH adjusted to 7.3 and mOsm to 290. Experiments were performed at room temperature (~22℃ using a MultiClamp700B amplifier (Axon Instruments) and Digidata 1440A digitizer under control of Clampex 10 software (Molecular devices). EPSCs were elicited by 0.5ms depolarizations from -70mV to 30 mV. RRP size was assessed by application of hypertonic sucrose (500 mM for 7 s)^6, 7^ using a piezo-controlled barrel application system (Perfusion Fast-Step, Warner Instruments). For mEPSC recordings in mass cultures, 1 μM tetrodotoxin was added to the external solution to block spontaneous action potential-driven activity. Only cells with an access resistance < 15MΩ (80% compensated) and leak current of <300 pA were included. Cells with an amplitude >20nA were excluded from analysis, as sufficient voltage clamp could not be assured in those cases.

*Analysis electrophysiological data.* Offline analysis was performed using custom-written programs^8^ in Matlab (Mathworks). Experimental conditions were blinded during analysis. In all figures, stimulation artefacts have been removed. For evoked release during 20 Hz stimulation, total charge was calculated by integrating the current from the end of the artefact until the start of the next artefact. Synchronous train release was separated from asynchronous release by subtracting the standing current at the start of the EPSC and integrating the first 25 ms of the EPSC, referred to as the synchronous window^9^. Asynchronous charge was determined by subtracting the synchronous charge from the total charge. Back-extrapolation of linear fits to the last 15 pulses of cumulative charge plots (Fig 2M, Fig 4N) were used to estimate the RRP (y-intercept) and recruitment rate (slope)^10^. The RRP estimate from synaptic responses to hypertonic sucrose solution was calculated by integrating the current within a 3s-window starting at the solution switch.

*Fitting procedures.* EPSC decays were fitted on smoothened data over a 350ms interval, starting after 2.5% of the maximum EPSC amplitude was recovered. Extra weights were added to the first 150ms to ensure correct fits during the steepest decay phases. Baseline current was determined as the mean current during the last 20ms of the fitting interval, and subtracted before fitting the data by least squares with a bi-exponential decay function without offset. Asynchronous charge was determined by integrating the slow component of the bi-exponential fit, and synchronous charge was calculated as the sum of the integrated current during the rising phase and the integral of the fast component. Tail current kinetics were determined by fitting a mono-exponential decay function with offset to the smoothened current recorded during a 2.75s interval starting at 10% amplitude recovery following 20Hz train stimulation.

*mEPSC detection.* Detection of mEPSCs was performed semi-automatically using custom-written software. Initially, potential events were identified based on a steep rising phase using an algorithm similar to^11^ followed by exponential decay, but the final selection of events was based on visual inspection. Event onset was defined as the last point before the event peak where the majority of data points in a trailing window were >-3pA/ms. Amplitude was determined by subtracting the current at event onset from the local peak current. Events with amplitudes < 4pA were excluded. mEPSC frequencies were analyzed over an interval of 45s, or until at least 200 events were detected within two separate intervals in the trace.

**Statistical Analysis**

Statistical significance was determined using student’s t-tests, with Satterthwaite’s correction for the degrees of freedom in case Levene’s test indicated unequal variances. Empirical vs theoretical cumulative distribution function (cdf) plots and Kolmogorov-Smirnov tests were used to assess normality. When normality could not be assumed, Mann-Whitney *U* tests were used. Sholl data was analyzed on log_10_(y+1)-transformed data using a mixed-effect model with sholl radius as repeated measure and condition as independent measure. Determination of the sample sizes was based on previous similar experiments^9, 12^. At least three independent cultures were used for each experiment. Reported effect size estimates are: Cohen’s d for t-tests, Wilcoxon’s r for Mann-Whitney U tests and η^2^ for linear models^13^. Boxplots have Tukey-style whiskers. Error bars of bar plots represent the SEM. All statistical tests were performed in Matlab (Mathworks), except for planned contrasts in ANOVA’s, which were done in SPSS (IBM).

**Data availability**

The datasets generated and analysed during the current study are available from the corresponding author on reasonable request.

**References**

1. Meijer M, Rehbach K, Brunner JW, Classen JA, Lammertse HC, van Linge LA *et al.* A single-cell model for synaptic transmission and plasticity in human iPSC-derived neurons. *Cell reports* 2019; **27**(7)**:** 2199-2211. e2196.

2. Bacaj T, Wu D, Yang X, Morishita W, Zhou P, Xu W *et al.* Synaptotagmin-1 and synaptotagmin-7 trigger synchronous and asynchronous phases of neurotransmitter release. *Neuron* 2013; **80**(4)**:** 947-959.

3. Hoogstraaten RI, van Keimpema L, Toonen RF, Verhage M. Tetanus insensitive VAMP2 differentially restores synaptic and dense core vesicle fusion in tetanus neurotoxin treated neurons. *Scientific Reports* 2020; **10**(1)**:** 10913.

4. Schindelin J, Arganda-Carreras I, Frise E, Kaynig V, Longair M, Pietzsch T *et al.* Fiji: an open-source platform for biological-image analysis. *Nature methods* 2012; **9**(7)**:** 676-682.

5. Schmitz SK, Hjorth JJ, Joemai RM, Wijntjes R, Eijgenraam S, de Bruijn P *et al.* Automated analysis of neuronal morphology, synapse number and synaptic recruitment. *Journal of neuroscience methods* 2011; **195**(2)**:** 185-193.

6. Rosenmund C, Stevens CF. Definition of the readily releasable pool of vesicles at hippocampal synapses. *Neuron* 1996; **16**(6)**:** 1197-1207.

7. Schotten S, Meijer M, Walter AM, Huson V, Mamer L, Kalogreades L *et al.* Additive effects on the energy barrier for synaptic vesicle fusion cause supralinear effects on the vesicle fusion rate. *Elife* 2015; **4:** e05531.

8. Huson V, Cornelisse LN. viewEPSC. 85b8899 edn2019.

9. Huson V, van Boven MA, Stuefer A, Verhage M, Cornelisse LN. Synaptotagmin-1 enables frequency coding by suppressing asynchronous release in a temperature dependent manner. *Scientific reports* 2019; **9**(1)**:** 1-19.

10. Neher E. Merits and limitations of vesicle pool models in view of heterogeneous populations of synaptic vesicles. *Neuron* 2015; **87**(6)**:** 1131-1142.

11. Ankri N, Legendre P, Faber D, Korn H. Automatic detection of spontaneous synaptic responses in central neurons. *Journal of neuroscience methods* 1994; **52**(1)**:** 87-100.

12. Lammertse HCA, van Berkel AA, Iacomino M, Toonen RF, Striano P, Gambardella A *et al.* Homozygous STXBP1 variant causes encephalopathy and gain-of-function in synaptic transmission. *Brain* 2020; **143**(2)**:** 441-451.

13. Fritz CO, Morris PE, Richler JJ. Effect size estimates: current use, calculations, and interpretation. *Journal of experimental psychology: General* 2012; **141**(1)**:** 2.
